# Minimally Invasive Mitral Valve Repair and Coronary Artery Bypass Graft by a Periareolar Approach

**DOI:** 10.1016/j.atssr.2025.07.007

**Published:** 2025-07-30

**Authors:** Hemn Abdulrahman Abdullah, Darya Nadir Saeed, Abdullah Hayder Flaih

**Affiliations:** 1 Cardiovascular and Thoracic Surgery, Shar Hospital, Erbil, Iraq; 2 Department of Biology, College of Sciences, Salahaddin University-Erbil, Erbil, Iraq

## Abstract

Use of coronary artery bypass for mitral valve repair in ischemic mitral regurgitation remains controversial. We present a case of a 52-year-old man with significant ischemic mitral regurgitation and coronary artery disease who underwent mitral valve repair and coronary artery bypass grafting using a periareolar technique. The procedure was successful, with early extubation and discharge on postoperative day 4. This minimally invasive approach facilitated successful repair while reducing surgical stress and enhancing recovery. Postoperative echocardiography confirmed the mitral valve's competence. This case highlights the viability of the periareolar approach for combined mitral valve repair and coronary artery bypass grafting.

Degenerative mitral regurgitation, which is commonly caused by leaflet prolapse or chordal rupture, is the leading form of primary mitral valve disease in developed countries.^1^ Some studies suggest that adding mitral valve repair (MVR) to coronary artery bypass grafting (CABG) can improve heart function, as evidenced by higher peak oxygen consumption and left ventricular reverse remodeling compared with CABG alone.^2^ However, these functional improvements do not always result in long-term survival benefits.^3^ When MVR is performed alongside CABG, operative and long-term mortality rates both remain comparable to those of CABG alone. Research indicates that the 2 groups have similar survival rates across follow-up periods ranging from 1 to 10 years.^4^

A 52-year-old male patient with a body mass index of 24.2, a nonsmoker, and no history of chronic conditions presented with left-sided chest pain radiating to the left shoulder and arm. The pain was throbbing in nature, associated with shortness of breath, fatigue, paroxysmal nocturnal dyspnea, palpitations, orthopnea, and dizziness. Other systemic examinations were unremarkable. The patient was classified as New York Heart Association Functional Classification II and Canadian Cardiovascular Society Class II. The Research Ethics Committee of the Dr. Hemn Foundation’s research center approved the study (approval reference number 17, on May 2, 2025). Informed consent was obtained from the patient before proceeding.

Preoperative coronary angiography revealed a proximal intermediate lesion in the left main stem (LMS), a critical midbifurcational lesion, and a critical proximal lesion in the left circumflex artery (LCX) (Figure 1).

**Figure 1:**
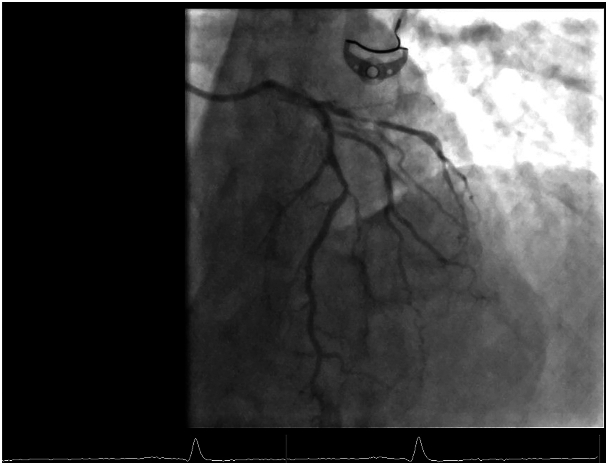
Preoperative coronary angiography shows coronary artery lesions.

Echocardiography showed severe posterior mitral valve prolapse with severe eccentric mitral regurgitation and a preserved left ventricular ejection fraction of 0.60. Results of p Preoperative laboratory investigations were largely unremarkable, except for mild microcytic hypochromic anemia (hemoglobin, 12 g/dL), low ferritin levels (10.9 μg/L), and severe vitamin D deficiency (6.21 ng/mL).

The patient was placed in a 20° semisupine position (Figure 2). General anesthesia was induced intravenously, and a double-lumen endotracheal tube was placed. A radial arterial catheter and right internal jugular central venous catheter were inserted for monitoring and drug administration. The radial artery was harvested using a LigaSure device (Medtronic) through 2 small incisions: one 3 cm above the wrist for distal ligation and the other just below the brachial artery bifurcation for proximal access. Dissection was done under direct vision without endoscopy or clips. The artery was flushed and stored in a plasma and papaverine solution.

**Figure 2:**
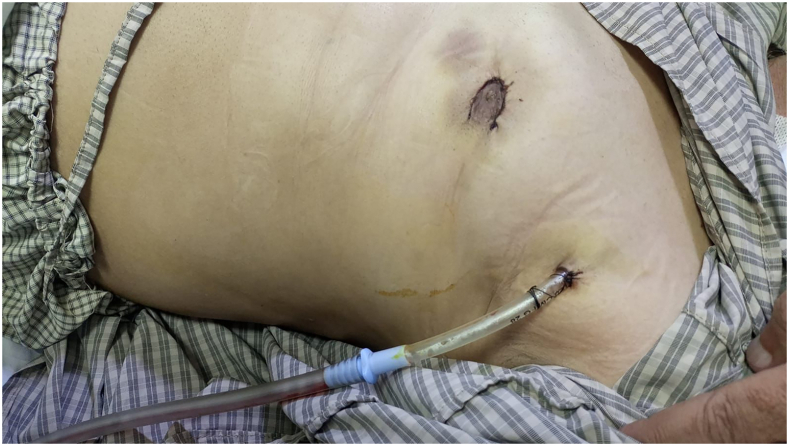
Postoperative periareolar incision and drainage.

A 1-cm crescentic left periareolar incision was made, medial to the areola, to facilitate access. The superficial fascia and pectoralis major were separated. The intercostal space was selected using apical 4-chamber echocardiographic imaging. A 12-mm camera port was inserted 2 spaces above the working incision. Heparin (5000 IU) was administered, and the left internal thoracic artery (LITA) was skeletonized from its subclavian origin to its bifurcation using a 30° Karl Storz 4K endoscopic camera. Rib retraction was avoided. Soft tissue and spinal retractors were used to enhance exposure.

For complete arterial revascularization, the loop technique was used: the LITA was anastomosed to the radial artery, creating a Y-graft to the left anterior descending and obtuse marginal arteries. After systemic heparinization, femoral-femoral cardiopulmonary bypass (CPB) was initiated using the Seldinger technique: right internal jugular (14F), femoral artery (17F), and femoral vein (25F) were cannulated with Medtronic cannulas. The pericardium was opened and suspended. On the left, the pericardium was incised 3 cm above and below the phrenic nerve, and stay sutures were exteriorized using a suture catcher. Vessel loops were placed on the inferior vena cava and superior vena cava for exposure.

The ascending aorta was dissected, and an antegrade cardioplegia cannula was inserted. A Chitwood cross-clamp was placed through the scope port, and del Nido cardioplegia was administered. CPB and aortic cross-clamp times were 180 and 145 minutes, respectively. Bicaval snaring was performed, and a right atriotomy extended from the appendage to the inferior vena cava. The interatrial septum was incised and retracted with stay sutures.

Intraoperative findings showed P2 prolapse due to ruptured chordae, consistent with fibroelastic deficiency. A limited triangular resection of P2 was performed. MVR used 5-0 Prolene (Ethicon) in continuous interlocked sutures and 3-0 Ethibond (Ethicon) for annuloplasty. A semirigid SJM annuloplasty band (St. Jude Medical) was implanted. An anterolateral commissural advancement stitch (“magic stitch”) improved coaptation (6-7 mm confirmed), with ink testing ensuring closure.

The septum and atriotomy were closed with 3-0 and 4-0 Prolene (Ethicon), respectively. LITA-to-left anterior descending and radial-to-obtuse marginal anastomoses were completed with 7-0 Prolene. After 15 minutes of reperfusion, rewarming, and air removal under transesophageal echocardiography, the patient was weaned from CPB uneventfully. Transesophageal echocardiography confirmed no mitral regurgitation.

The patient was transferred to the intensive care unit on inotropic support, extubated in 3 hours, spent 17 hours in the intensive care unit, and was discharged on day 4 without complications.

## Comment

For patients with severe MR undergoing CABG, concurrent MVR or replacement is essential to optimize long-term outcomes. However, in cases of moderate MR, the necessity of simultaneous MVR with CABG remains controversial, and some studies suggest it may increase morbidity and mortality without significant clinical benefit.^4^ Although some cases of MR improve with CABG alone,^5^ our patient had severe MR with significant occlusions in the LCX and LMS, necessitating a combined surgical approach. A periareolar incision approach was used to optimize outcomes and minimize surgical trauma.

To achieve optimal results while minimizing surgical trauma, we used a periareolar incision, which enabled both MVR and CABG. This approach was selected due to its proven feasibility, cosmetic advantages, and potential to reduce postoperative pain and recovery time.^6^

In conclusion, this case highlights the efficacy of the periareolar approach for combined CABG and MVR in a patient with severe posterior mitral valve prolapse, severe eccentric MR, and LMS and LCX lesions. This strategy aimed to minimize surgical trauma, distress, and recovery time. The patient was discharged on postoperative day 4 without complications.
